# Evaluation of the Effects of Cold Plasma on Cell Membrane Lipids and Oxidative Injury of *Salmonella typhimurium*

**DOI:** 10.3390/molecules27030640

**Published:** 2022-01-19

**Authors:** Xiaoye Lv, Jun-Hu Cheng

**Affiliations:** 1 School of Food Science and Engineering, South China University of Technology, Guangzhou 510641, China; hljin2008@126.com; 2 Academy of Contemporary Food Engineering, South China University of Technology, Guangzhou Higher Education Mega Center, Guangzhou 510006, China

**Keywords:** cold plasma, *S. typhimurium*, membrane lipid oxidation, antioxidant enzyme

## Abstract

*Salmonella typhimurium* (*S. typhimurium)* is a major causative agent of foodborne illness worldwide. Cold plasma (CP) was used to inactivate *S. typhimurium* and to investigate the effect of CP on cell membrane lipids and oxidative injury of cells. Results indicated that the inactivation effect of CP on *S. typhimurium* was positively correlated with the treatment time and voltage. *S. typhimurium* was undetectable (total number of surviving colonies <2 log CFU/mL) after 5 min treatment with the voltage of 50 V. CP treatment caused damage to the cell membrane of *S. typhimurium* and the leakage of cell contents, and the relative content of unsaturated fatty acids in cell membrane decreased. Cell membrane lipids were oxidized; the malondialdehyde content increased from 0.219 nmol/mL to 0.658 nmol/mL; the catalase activity of *S. typhimurium* solution increased from 751 U/mL to 2542 U/mL; and the total superoxide dismutase activity increased from 3.076 U/mL to 4.54 U/mL, which confirmed the oxidative damage in *S. typhimurium* cell membrane caused by CP treatment. It was demonstrated that the potential application of plasma-mediated reactive oxygen species is suitable for destroying the structures of the cell membrane and ensuring the microbial safety of fresh food samples.

## 1. Introduction

*Salmonella typhimurium* (*S. typhimurium)* is a major causative agent of foodborne illness worldwide, resulting in approximately 155,000 deaths each year [1]. The current research on food safety of *S. typhimurium* mainly focuses on milk, eggs and poultry meat products. With the rise of green agriculture, organic fertilizers are widely used in the cultivation of vegetables and fruits, especially for directly contacting the soil. Fruits and vegetables, such as lettuce, strawberries, cucumbers, etc., are easily contaminated by *S. typhimurium.* These fruits and vegetables are mainly eaten directly, which is more likely to lead to the contamination with *S. typhimurium* and threaten human health. Cold plasma (CP), as an innovative and environmentally friendly technology, shows great potential for decontamination of fresh-cut fruits and vegetables [2,3] .

As for the inactivation mechanism of microorganisms, using CP treatment might be divided into two aspects: physical damage and biochemical damage [2]. The physical sterilization mechanism of CP may be similar to the sterilization mechanism of pulsed electric field, which means that the formation of perforations in the cell membrane causes the contents to leak and ultimately leads to cell inactivation [4]. The current popular biochemical mechanism of plasma sterilization is that plasma discharge in oxygen-containing gas produces highly reactive substances, including reactive oxygen species (ROS), reactive nitrogen species (RNS), ultraviolet radiation, high-energy ions and charged particles [5]. Furthermore, these active materials react with water to produce many short-lived active ingredients, such as OH, ^1^O_2_ and long-lived active materials, such as H_2_O_2_, which are considered to be important deactivators [6]. It is generally believed that cellular components, including cell wall, cell membrane, intracellular proteins and DNA, are the main damage targets of these exogenous ROS [1]. However, due to the complexity of CP treatment and microbial systems, the mechanism of plasma sterilization has so far been difficult to describe clearly. In the study of Mai-Prochnow et al. [1], it was explained in detail that the cell wall structure of Gram-negative bacteria and Gram-positive bacteria was different, and the sensitivity to ROS was also different. Han et al. [7] compared two different inactivation mechanisms and found that cell rupture and slight DNA damage were the main causes for inactivating *Escherichia coli* (*E. coli*); on the contrary, the inactivation of *Staphylococcus aureus* (*S. aureus*) was mainly due to the intracellular damage caused by a large amount of exogenous ROS. Under the action of ROS, the unsaturated fatty acids of cell membrane lipid undergo lipid hydrogen peroxide reaction, which might lead to the destruction of cell membrane structure [8].

However, it is unclear whether the membrane lipid oxidation is caused by the oxidative stress of exogenous ROS on bacteria. In addition, it is necessary to further explore the influence of exogenous oxidative stress of ROS on cell survival. Thus, the objective of the present study was to investigate the sterilization effect of dielectric barrier discharge (DBD) plasma treatment time, voltage and storage time on *S. typhimurium* and to explore the effect of CP on lipids and oxidative injury of *S. typhimurium* based on the cytoplasmic membrane permeability, fatty acid composition and lipid peroxidation. Additionally, the effects of CP on the major antioxidant enzyme activities (catalase, superoxide dismutase) were also determined to prove the stress behavior of *S. typhimurium* in response to CP treatment.

## 2. Materials and Methods

### 2.1. Bacterial Strains and Culture Conditions

*S. typhimurium* ATCC 14028 was purchased from Guangdong Microbial Food Safety Engineering Technology R&D Center (Guangzhou, China). Tryptic soy broth with 0.6% yeast extract (TSB-YE) was adopted to activate the freeze-dried strain and then maintained as frozen stocks in tubes at −80 °C with 50% (*v*/*v*) of glycerol. Before the experiment, the strain was stored at −80 °C and inoculated with fresh tryptic soy agar (TSA) to obtain a single colony. The activated single colony of *S. typhimurium* cultured in TSA medium was placed in 100 mL of 0.6% TSB-YE and cultured at 37 °C and 150 r/min for 4 h until the exponential phase (OD_600_ nm = 0.40–0.50) was achieved. Then, the bacteria were washed and re-suspended in sterile cold (4 °C) phosphate-buffered saline (0.01 mol/L PBS, pH = 7.4), and the concentration of the suspension was adjusted to approximately 10^8^ CFU/mL. All the culture materials and chemicals were obtained from Guangzhou Chemical Reagent Factory (Guangzhou, China).

### 2.2. Plasma System Configuration

**Figure 1:**
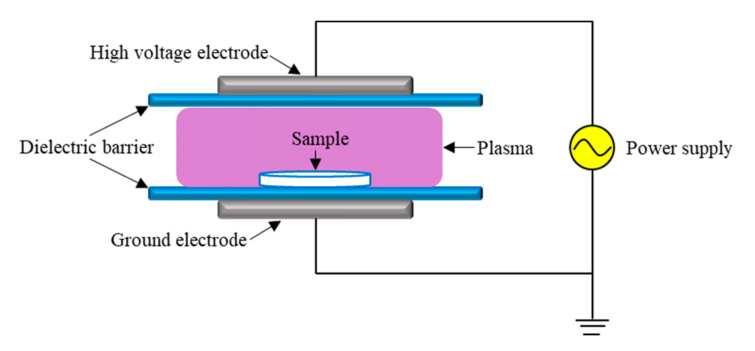
Schematic diagram of DBD atmospheric pressure cold plasma.

The *S. typhimurium* suspension was exposed to a DBD plasma system, which was jointly designed by South China University of Technology (Guangzhou, China) and Nanjing Suman Plasma Technology Co. Ltd. (Nanjing, China). A 5 mm dielectric plate was used as a barrier electrolyte to cover the lower part of the high voltage electrode on the sample processing table. During the treatment, the distance between the two electrodes was controlled to be 5 cm; the frequency was 5–20 kHz; and the input voltage was 40–70 V. *S. typhimurium* suspension was directly exposed to the air medium, setting the treatment time according to the experimental needs. Lastly, the treated suspension was placed in bacteria liquid in the refrigerator at 4 °C. Figure 1 shows the schematic diagram of DBD atmospheric pressure cold plasma.

### 2.3. Physicochemical Properties Measurement

The temperature of samples (10 mL) after DBD plasma treatment was immediately recorded by using an infrared imaging camera (FLIR E5, FLIR Systems AB, Taby, Sweden), with a resolution of 120 × 90 pixels and a wavelength range of 7.5–13 μm (Pan et al., 2019). Meanwhile, a pH meter (Testo 205, Testo AG, Lenzkirch, Germany) was used to measure the pH values of samples (20 mL), and electrical conductivity was measured with electroconductivity meter (DDS-307, Shanghai, China).

### 2.4. Microbiological Enumeration

An amount of 100 μL of bacterial suspension after plasma treatment was diluted and spread uniformly on TSA agar plates and incubated at 37 °C for 24–48 h. The inactivation ability of plasma was evaluated by the formula: Log Reduction = Log_10_(N/N_0_) [9]. N_0_ represents the initial number of colonies forming units, and N represents the number of colonies forming units after treatment. Meanwhile, 100 μL of the suspension was used to determine the inactivation ability of plasma-mediated ROS during storage time at 4 °C for different days after CP treatments.

### 2.5. Cell Membrane Integrity

UV absorbances at 260 and 280 nm are generally used to quantify the concentration of DNA and protein, respectively, and this method can also detect the loss of bacterial cell membrane integrity and the release of intracellular proteins and nucleic acids after CP treatment [10]. The end of the exponential phase of *S. typhimurium* in TSB medium was adjusted to OD_600_ of 1.0. Cells were centrifuged at 4000× *g* for 5 min, supernatant was discarded, and pellet was washed twice by sterilized PBS. A quartz cell holding the supernatant of samples was used to determine the UV absorbances at 260 nm and 280 nm. All the measurements were taken in triplicates with UV-1800 spectrophotometer (Shimadzu Co., Kyoto, Japan).

### 2.6. Membrane Permeability Observation

The permeability of *S. typhimurium* cell membrane was determined by confocal laser scanning microscopy (CLSM) (TI-EAI, NIKON, Japan). Amounts of 0.1 mL of PI solution (100 mmol/L) and 4 mL of plasma-treated *S. typhimurium* suspension were mixed and incubated at 37 °C for 30 min in the dark. Samples after reaction were washed and re-suspended in sterile cold PBS (0.01 mol/L, pH = 7.4, 4 °C). The parameters of the CLSM were as follows: excitation wavelength of 362 nm and emission wavelength of 432 nm [11].

### 2.7. Membrane Fatty Acid Composition Determination

According to the method of Wang et al. [12], the pellets after plasma treatment were obtained by centrifugation (8000 × *g*, 15 min, 4 °C). Samples washed three times with PBS were re-suspended in 1.0 mL of methanol: 3.75 mol/L NaOH (1:1, *v*/*v*) mixture solution, pipetted into test tubes and heated at 100 ± 1 °C for 5 min, after being thoroughly mixed again, and were then treated with boiling water for 25 min. Afterward, 2.0 mL methylation solution (32.5 mL 6.0 mol/L HCl and 27.5 mL methanol) was added to the cooled sample. The samples were then vortexed for 7.5 ± 2.5 s and placed in a water bath at 80 ± 1 °C for 10 min. The test tubes were cooled before the addition of 1.25 mL of hexane: methyl tert-butyl ether (1:1, *v*/*v*) mixture solution to each tube, and the tubes were tumbled for 10 min. The lower phase was then removed, and 3 mL of 0.3 mol/L NaOH was added to each tube. After tumbling for further 5 min, waiting for obvious layering, 2/3 of the upper phase was pipetted into a gas chromatography (GC) vial, which was stored at −80 °C until analysis.

The membrane fatty acid composition was analyzed by gas chromatography-mass spectrometry (GCMS, QP2010, Shimadzu Co., Tokyo, Japan). The identification results of methyl esterified fatty acids were expressed as the relative percentage of each fatty acid, and the computer calculated the proportion of the area under each fatty acid peak to the total area of all the calculated fatty acid peaks.

### 2.8. Membrane Lipid Peroxidation Detection

The thiobarbituric acid (TBA) method was used to determine the degree of oxidation of plasma-treated cell membrane lipids and the control. After repeated freezing and thawing, the sample was centrifuged to remove the bacteria and retain the supernatant. An amount of 1 mL of the supernatant was transferred to a 10 mL centrifuge tube, and 2 mL of 0.6% TBA solution was added. After mixing, the tube was placed in a water bath at 95 ± 1 °C for 20 min and then quickly cooled in an ice bath for 5 min. Finally, the samples were detected by spectrophotometer (UV-1800, Tokyo, Japan) at 532 nm and 600 nm. Equation (1) was used to calculate the oxidative damage in *S. typhimurium* cells induced by plasma treatment:

MDA concentration (nmol/mL) = [(A_532_ − A_600_)/1550] × 10^3^ × 3 (1)

### 2.9. Morphological Changes Analysis

The morphological changes of *S. typhimurium* were observed by scanning electron microscope (SEM) (JSM-6360LV, Tokyo, Japan). First, the plasma-treated and the control suspensions were centrifuged (4000× *g*, 5 min), and the collected bacterial pellets were re-suspended in 2.0 mL fixative of paraformaldehyde: glutaraldehyde (2%:2.5%, *w*/*w*) mixture solution for 12 h at 4 °C. Subsequently, the fixative was discarded by centrifugation, and the samples were washed with 0.1 mol/L PBS three times. Second, the samples were dehydrated by graded ethanol from 30–95% (30, 50, 70, 85 and 95%) for 15 min, respectively, and finally dehydrated with 100% ethanol twice for 20 min. The ethanol-dehydrated cells were then substituted in isoamyl acetate twice for 20 min. After the above steps, the samples were placed on a silicon wafer and freeze-dried for 24 h in a vacuum environment. Finally, all samples were performed for gold sputtering and then observed by SEM.

### 2.10. Intracellular ROS Level

2′,7′-dichloro-dihydro-fluorescein diacetate (DCFH-DA) obtained from Sigma-Aldrich Trading Co. Ltd. (Shanghai, China) can penetrate the cell membrane freely. The DCFH-DA was converted into non-fluorescent DCFH in the cell. DCFH can be further oxidized by intracellular ROS into highly fluorescent DCF. It can be considered that the content of DCF is directly proportional to the concentration of intracellular ROS formation. Therefore, DCFH-DA has been applied to detect the intracellular ROS levels [13]. An amount of 100 μL of DCFH-DA at a concentration of 20 mM was added to 1 mL of the treated bacterial suspension and incubated for 30 min at 37 °C in the dark.

### 2.11. Activity of Antioxidant Enzymes

The activity of antioxidant enzymes, including catalase (CAT) and superoxide dismutase (SOD) of samples, was analyzed following the instructions of Assay Kit bought from Yuanye Biological Technology Co., Ltd. (Shanghai, China). Finally, the samples were detected by ELIASA (Synergy2, BioTek, Winooski, VT, USA) at 530 nm and 405 nm, respectively, to determine the activity of antioxidant enzymes.

### 2.12. Statistical Analysis

OriginLab 8.0 software (OriginLab Inc., Northampton, MA, USA) was applied to perform the statistical analysis. The differences were further evaluated by the least significant difference test (LSD) at the *p* < 0.05 level based on analysis of variance (ANOVA) by SPSS software (version 20.0) (IBM Analytics, New York, NY, USA). Each experiment was repeated in triplicate, and averaged data were reported.

## 3. Results and Discussion

### 3.1. Physical and Chemical Characteristics Analysis during Plasma Treatment

**Figure 2:**
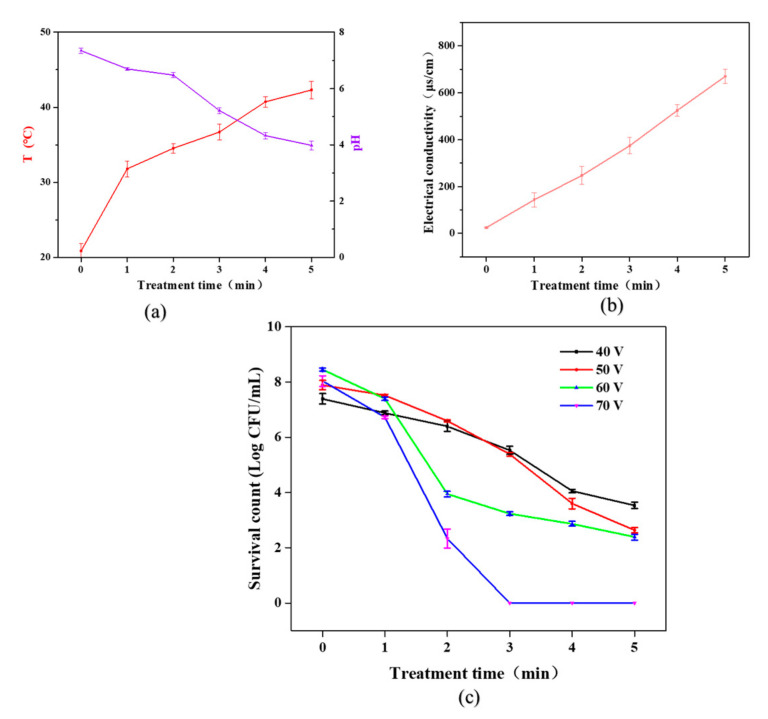
Effects of cold plasma treatment on *S. typhimurium* suspension. (**a**) Temperature changes; (**b**) electrical conductivity changes; (**c**) total number of *S. typhimurium* colonies changes.

As shown in Figure 2a, during DBD plasma treatment, the average temperature of *S. typhimurium* bacteria liquid increased with the increase in treatment time. However, when the treatment time was 5 min, the liquid temperature was 42.3 °C. Therefore, the lethal effect of temperature changes on *S. typhimurium* was insignificant. The pH gradually decreased with the increase in treatment time; especially after 3 min treatment, the pH of the bacterial suspension dropped to about 4.73 at 4 min. Existing studies have shown that the low pH of the solution played an important role in the sterilization effect caused by the gas-phase discharge plasma operating at the gas–liquid interface [14]. The study reported by Pan et al. [15] showed that the pH of the solution stabilized at 7.13 after being treated with a mixed gas of argon and oxygen (Ar:O_2_ = 98:2) for 8 min. This also demonstrated that the pH of the solution using inert gas as a gas source did not drop significantly. The conductivity of the CP-treated *S. typhimurium* bacterial solution is shown in Figure 2b. With the increase in treatment time, the conductivity of *S. typhimurium* bacteria solution continued to rise from 25 ms/cm to 670 ms/cm, which further demonstrated that a large number of active particles were accumulated in the liquid phase during DBD plasma treatment.

### 3.2. Injured Cell Analysis

Figure 2c illustrates the inactivation of *S. typhimurium* by CP treatment at different voltages and times. It was noted that with the increase in CP treatment time (0–5 min), the total number of surviving colonies gradually decreased. When the input voltage was 40 V, the total number of colonies was only reduced by 3.3 log CFU/mL after 5 min treatment. When the input voltage was higher than 60 V, with the processing time of 2 min, *S. typhimurium* was essentially undetectable. Excessive input voltage produced intense electric spark discharge, which to some extent caused serious physical damage to the bacteria, accelerating the rupture of the cell membrane of *S. typhimurium*.

**Figure 3:**
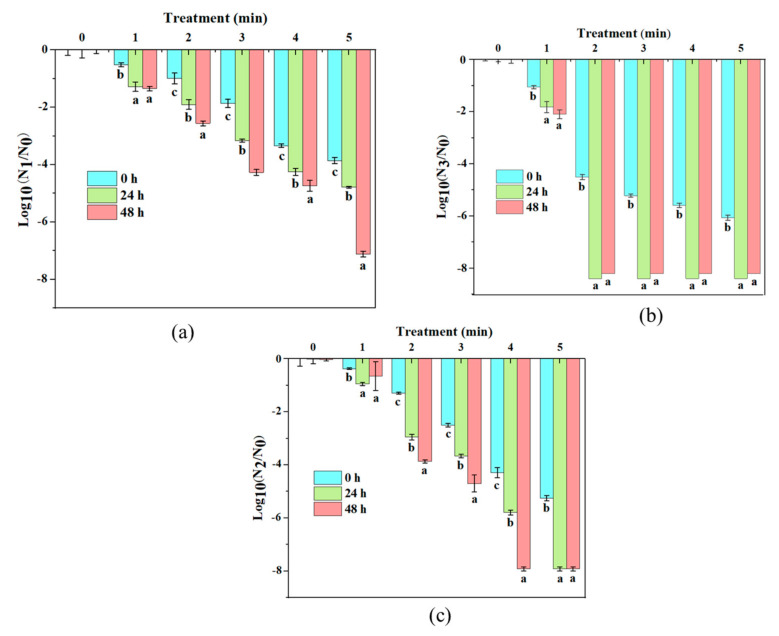
Inactivation of *S. typhimurium* at different storage time after plasma treatment: (**a**) 40 V, (**b**) 50 V, (**c**) 60 V. Different letters of each treatment time indicate the significant difference (*p* < 0.05).

Under a certain input voltage and processing time, with the prolonged storage period of the bacterial suspension at 4 °C, the inactivation effect of *S. typhimurium* was more obviously illustrated in Figure 3. When the input voltage was 40 V, with the increase in treatment time (0–5 min), the reduction of log10 cycles dramatically increased to 7 log CFU/mL, and *S. typhimurium* reached undetectable status. In addition, it was noticed that, compared with the second 24 h, the first 24 h storage period achieved a more obvious inactivation rate. For example, when the input voltage was 40 V and the treatment time was 2 min, during the first 24 h storage period, the reduction was 1 log CFU/mL, while during the second 24 h storage, the reduction was 0.5 log CFU/mL. This may be due to the reaction between the remaining reactive species of dead cells and the bactericidal active substance in the bacterial solution. Thereby, it is a good way of prolonging the time for the active substance to further inactivate the bacteria [7]. This proved that the active substances accumulated in the liquid by the plasma treatment, such as OH, ^1^O_2_, O_2_ and H_2_O_2_, exhibited an oxidative lethal effect on cells. Pan et al. [15] and Ziuzina et al. [16] also confirmed the existence of this phenomenon during their studies.

### 3.3. Cell Membrane Integrity and Permeability Analysis

**Figure 4:**
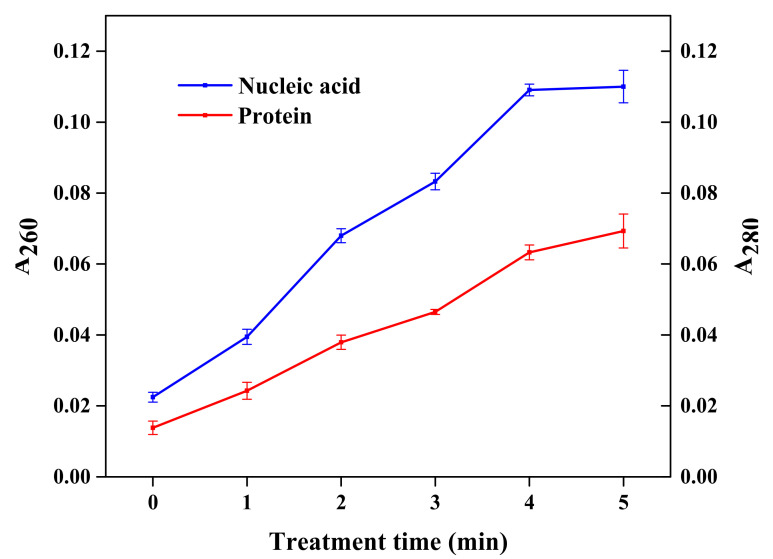
Effect of cold plasma treatment on the concentration of nucleic acid and protein in *S. typhimurium* suspension.

The values for absorbance at 260 nm (DNA) and 280 nm (protein) are usually used to monitor membrane integrity. The concentration of DNA and protein is generally proportional to the absorbance value, which can promote a deeper understanding of plasma-mediated cell leakage. The values for absorbance at 260 nm (A_260_) and 280 nm (A_280_) for supernatants of *S. typhimurium* treated in PBS are depicted in Figure 4. With the increase in plasma treatment time, the values of A_260_ and A_280_ of *S. typhimurium* suspension increased, indicating that the concentration of DNA and protein increased, and the integrity of *S. typhimurium* was continuously lost. An analogous effect was also observed in *E. coli* [7]. However, according to the study of Pan et al. [15], they indicated that there were no considerable leakage levels of DNA and protein of *L. monocytogenes* occurring with prolonged treatment time. This indicated that the plasma inactivation mechanism for Gram-negative bacteria and Gram-positive bacteria was different. Gram-negative bacteria did not have a tightly cross-linked cell wall structure of peptidoglycan, and the plasma oxidative damage ruptured the cell membrane, resulting in bacterial inactivation.

**Figure 5:**
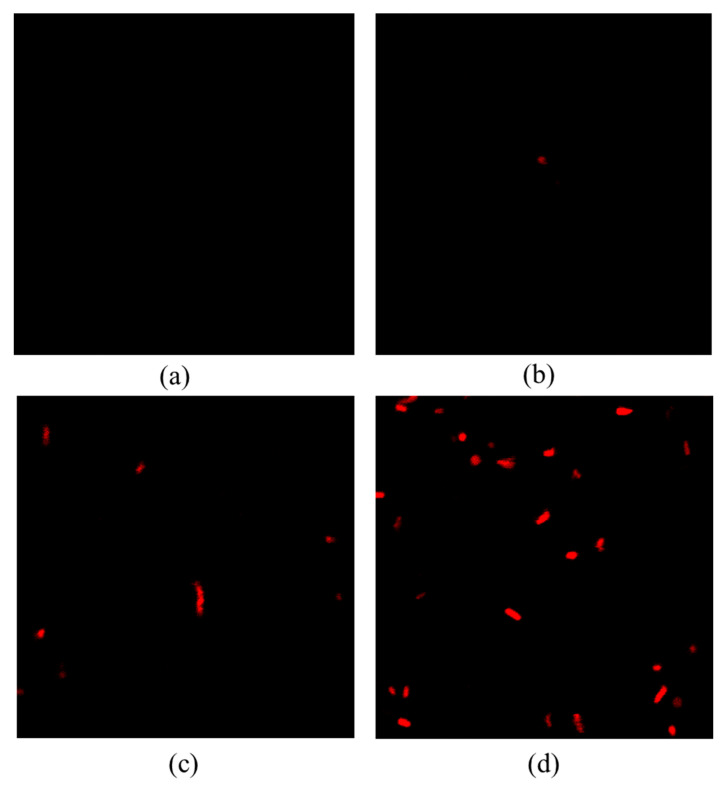
Effect of cold plasma treatment time on the permeability of *S. typhimurium*. (**a**) Control; (**b**) DBD-treated for 1 min; (**c**) DBD-treated for 3 min; (**d**) DBD-treated for 5 min.

In order to obtain a deeper understanding of plasma-mediated cell membrane damage, the number of red fluorescent cells can be used to intuitively determine the degree of damage to bacterial cells by PI staining technology [17]. Figure 5 shows the effect of different time of DBD plasma treatment on the permeability of *S. typhimurium* cell membrane. As the treatment time increased, the number of fluorescent cells gradually increased, and the number of inactivated bacteria also gradually increased, indicating that the number of fluorescent cells was positively correlated with the number of inactivated bacteria. This indicated that the greater the permeability of the cell membrane, the severer the cell membrane damage. Dolezalova et al. [18] also reported the application of PI dyes in experiments to verify plasma damage to *E. coli* cell membrane. Therefore, it can be concluded that the cell membrane was oxidized and destroyed, and the permeability of the cell membrane increased. The formation of pores in the cell membrane and the outflow of the contents of the cell would finally lead to the inactivation of bacteria.

### 3.4. Effect of CP on Lipid Composition and Oxidative Injury

The lipid composition of the cell membrane plays an important role in maintaining the integrity of the cell membrane [19]. The cytoplasmic membrane is the outer permeable barrier of the cell, coordinating a variety of physiological processes and stress response, and its function is determined by their fatty acids and its membrane protein compositions. The composition of the membrane lipid of *S. typhimurium* under the same culture conditions is already relatively clear [20]. Table 1 shows the influence of CP treatment on fatty acid composition of *S. typhimurium*. The relative content of unsaturated fatty acids (UFA) (C16:1, C18:1 and C18:2) in the cell membrane of *S. typhimurium* decreased significantly, and the relative content of cyclic fatty acids also decreased slightly. There was also a significant decrease in the ratio of UFA to saturated fatty acid (SFA), from 0.310 to 0.089 after CP treatment, indicating that UFA in the cell membrane could be oxidized into other substances by ROS. These changes were predominantly ascribed to a decrease in C18:1 and C18:1. In particular, polyunsaturated fatty acids (PUFA) were considered to be the most susceptible UFA for oxidation. In the lipid peroxidation chain reaction, PUFA is oxidized by ROS to form a fatty acid free radical (L), which is finally oxidized to lipid hydrogen peroxide (LOOH) [4].

**Figure 6:**
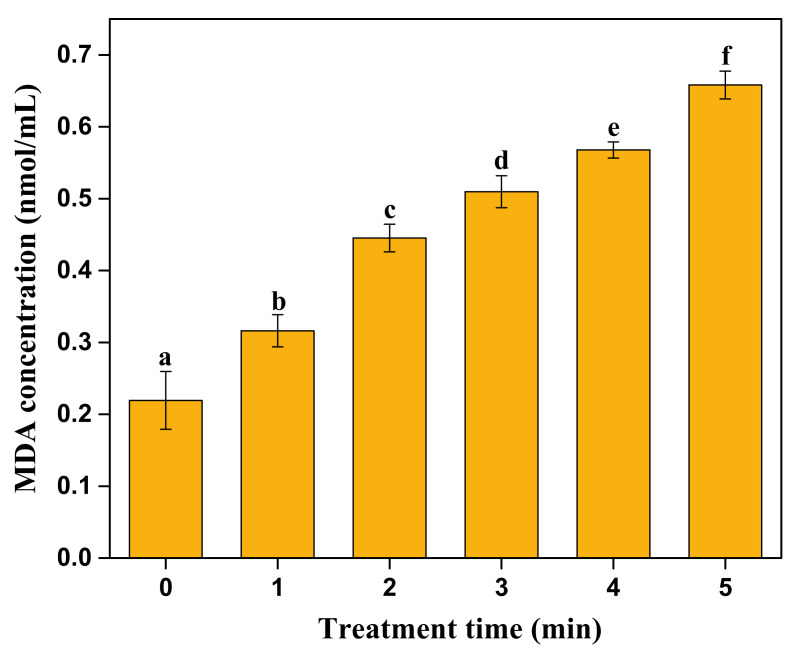
MDA changes of *S. typhimurium* suspension after cold plasma treatments. Different letters indicate the significant difference (*p* < 0.05).

In the lipid peroxidation chain reaction, malondialdehyde (MDA) is one of the products of this process, which can directly reflect the degree of membrane damage [4]. The content of MDA changes after CP treatment is depicted in Figure 6. With the increase in plasma treatment time, the MDA content in the bacterial suspension showed a continuous upward trend with a significant difference (*p* < 0.05). Similarly, Dolezalova et al. [18] also illustrated that the MDA concentration of the bacillus suspension increased with the increase in treatment time. Therefore, a decrease in unsaturated fatty acids in the cell membrane of *S. typhimurium* indicated an increase in oxidative pressure caused by exogenous ROS, which resulted in the appearance of oxidative damage in the cell membrane of *S. typhimurium.*

### 3.5. Cell Morphology Analysis

**Figure 7:**
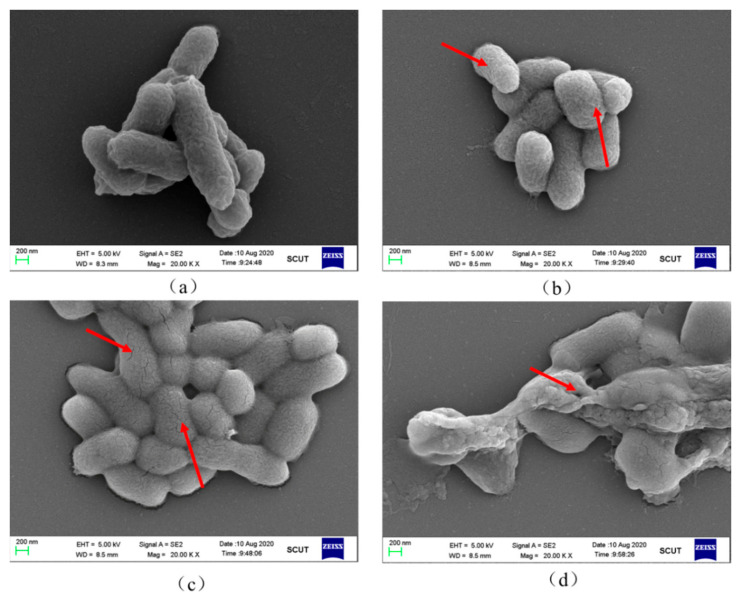
Effect of cold plasma treatment on the morphology of *S. typhimurium.* (**a**) Control; (**b**) DBD-treated for 1 min; (**c**) DBD-treated for 3 min; (**d**) DBD-treated for 5 min. Red rows indicate the modifications in cell morphology and cell debris.

The effect of different plasma treatment times on *S. typhimurium* cell morphology is depicted in Figure 7. Figure 7a illustrates the morphology of *S. typhimurium* without plasma treatment, maintaining the normal cell shape with no shrinkage. However, *S. typhimurium* visibly shrank, and its length was reduced after CP exposure for 1 min. As for the reasons, when *S. typhimurium* was in a non-lethal oxidizing environment, the bacteria reduced the surface area, and the energy metabolism activities were required for survival to be forced or automatically transformed into a form that was more conducive to survival [2]. This phenomenon of bacteria shrinking after being treated with plasma for a short time was reported in the studies of *E. coli* [21], *C. freundii* [22] and *S. aureus* [7]. Dezest et al. [23] also observed similar shrinkage changes of *E. coli* from rod shaped to spherical, but they attributed this change to the physical effects of charged ions and electric fields during plasma treatment, rather than the oxidative pressure generated by exogenous ROS. However, Joshi et al. [24] found that after plasma treatment and storage for 24 h, *E. coli* contracted from a typical rod shape into a shrinking chlorella cell. In addition, in the absence of an electric field, Liao et al. [4] also observed the cell shrinkage of *S. aureus* treated with plasma activated water. The above studies indicated that the oxidative pressure produced by ROS was the main factor in bacterial shrinkage. When the treatment time reached 3 min, obvious cracks appeared on the surface of *S. typhimurium* cells, which indicated that the contraction of the bacteria could not adapt to the increasingly strong oxidizing environment, and the bacterial cell membrane was obviously damaged. As shown in Figure 7d, an obvious phenomenon was clearly observed, and the bacteria could no longer maintain the original bacterial form. A large amount of intracellular material leaked after being exposed to plasma for 5 min. This phenomenon corresponded to the correlation analysis of cell permeability, integrity, cell membrane fatty acid and MDA content. Therefore, based on the above discussion, it can be inferred that with the continuous enhancement of the ROS oxidation environment induced by DBD plasma, the higher the degree of oxidative damage to the cell membrane of *S. typhimurium*, the higher the inactivation rate increase.

### 3.6. Intracellular ROS Level

**Figure 8:**
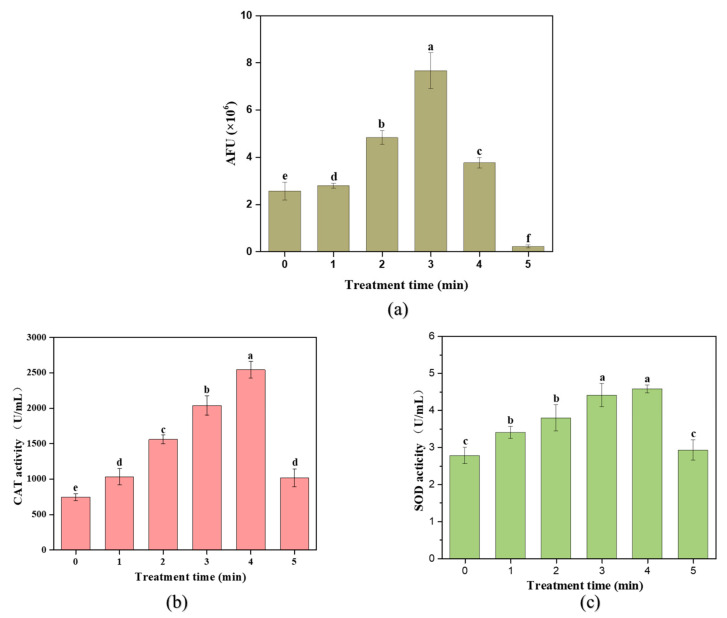
Effects of cold plasma treatment time on the *S. typhimurium* cell. (**a**) Changes of intracellular ROS in cells; (**b**) changes of CAT activity; (**c**) changes of SOD activity. Different letters indicate the significant difference (*p* < 0.05).

In the plasma gas-liquid discharge process, the short-lived reactive species, such as OH and O_2_^−^, are the main active components for inactivating bacteria, while H_2_O_2_ possesses the continuous residual bactericidal effects [6]. In order to further verify the reasons for bacteria being inactivated mainly by oxidative damage, it is necessary to measure the level of total ROS in cells. Figure 8a shows the DCF fluorescent signals in cells after plasma treatment for different times. When the treatment time was from 0 min to 3 min, DCF fluorescent signals of cells increased significantly, which indicated that a large amount of ROS entered the *S. typhimurium* cell during the plasma treatment process, and the longer the processing time, the more exogenous ROS accumulated in the cell. However, when the treatment time reached 4 min, the DCF fluorescent signals dropped sharply. After CP exposure for 5 min, the fluorescence signals were even lower than those of untreated *S. typhimurium*. The result agreed with the study reported by Pan et al. [15], who found that the level of ROS in *L. monocytogenes* cells after plasma treatment suddenly dropped from 52.94% to 42.18% with prolonged treatment for 8 min. There may be two reasons for this phenomenon. On one hand, as the plasma exposure time was prolonged, the ruptures of the cell membrane caused the leakage of intracellular components, including ROS, which corresponded to the analysis of cell membrane rupture after damage. On the other hand, due to the accumulation of exogenous ROS that damaged the various contents of the cell, including proteins, nucleic acids and other macromolecular organisms, the activity of the esterase protein was inactivated, and the DCFH-DA could no longer be catalyzed to DCFH. Thus, the corresponding highly fluorescent DCF greatly reduced [8]. In summary, plasma treatment caused the accumulation of exogenous ROS in the cell, and at the same time, ROS accumulated in the cell and attacked the biological macromolecules in the bacterial cell indiscriminately, further leading to the inactivation of bacteria.

### 3.7. Effect of CP on Antioxidant Enzyme Activity

During the persistent entering of external ROS to the cells, the regulation of the antioxidant enzyme system (CAT and SOD) within the bacterial cells is theoretically critical to cell survival [25]. Therefore, in order to further verify the oxidative stress effect of exogenous ROS on bacteria, it is necessary to evaluate the effect of plasma treatment on the enzyme activities of CAT and SOD in bacterial cells. The results showed that compared to the control sample, two antioxidant enzyme activity levels in the bacterial solution exhibited very significant differences. For instance, the CAT activity (Figure 8b) in the bacterial solution after plasma treatment for 4 min reached 2542 U/mL, which was more than three times that of the control sample. This indicated that the ROS generated by the plasma at the gas–liquid interface caused a significant upregulation of the CAT system inside the bacterial cell. Although the SOD activity (Figure 8c) in the treated bacterial solution gradually increased, the increase was significantly lower than that of CAT, only increasing from 3.076 U/mL to 4.54 U/mL. According to the result of Simoncicova et al. [26], similar changes were observed in the CAT enzyme activity of *A. flavus* after plasma treatment, which was significantly higher than the total SOD enzyme activity. This result might be related to the short-lived reactive species (O_2_^−^) that are less stimulating to the corresponding antioxidant enzyme system than the long-lived reactive species (H_2_O_2_) [6]. When the treatment time reached 5 min, the activities of CAT and SOD were significantly reduced. Roth et al. [27] also found that prolonged plasma treatment time resulted in a linear decrease in the CAT enzyme activity of *B. subtilis*. One possible reason was the structural change of the active center of the enzyme during the treatment process, leading to the inactivation of the enzyme [10]. The external morphology of the bacteria after 5 min treatment (Figure 7d) indicated that the external damage in cell membrane was serious, and the cell content leaked. These antioxidant enzymes were exposed to the external acidic strong oxidizing environment, and the active center of the enzyme was easily destroyed. It can be concluded that the accumulation of oxidative active substances produced by the plasma at the gas–liquid interface caused the bacteria to be at a severe oxidative stress state.

## 4. Conclusions

The correlation between DBD plasma processing parameters, such as input voltage and processing time and inactivation efficiency, was studied, and the effect of storage period on bacterial inactivation was explored. Results showed that the efficiency of plasma inactivation of *S. typhimurium* increased with the increase in input voltage and treatment time. In addition, plasma exposure with subsequent storage at 4 °C achieved further noticeable inactivation and confirmed the inactivation effect of plasma-mediated ROS on bacteria. The effects of CP on membrane integrity, fatty acid composition and lipid peroxidation were also investigated. CP treatment destroyed the integrity of *S. typhimurium* cells and altered the permeability of cell membrane. CP treatment resulted in oxidative injury of *S. typhimurium*, which was characterized by an increase in the content of MDA and a decrease in the value of UFA/SFA. The changes of total ROS in *S. typhimurium* and the regulation of the two main antioxidant enzyme activities verified that DBD plasma treatment caused oxidative stress in bacterial cells. These results suggested the contribution of plasma-mediated ROS in destroying the cell membrane of *S. typhimurium* and then leading to cell inactivation. More attention should be focused on the effects of CP treatment on other important substances that maintain the structure of cell membrane to reveal the destruction mechanism of ROS on cell membrane.

## Figures and Tables

**Table 1:** Effects of DBD plasma treatment on the fatty acid composition of *S. typhimurium* cell.

Fatty Acid Composition	Content (%)		
	Control	CP for 2 min	CP for 4 min
Saturated fatty acid (SFA)			
C12:0	2.13 ± 0.05 ^a^	1.82 ± 0.08 ^b^	1.61 ± 0.03 ^c^
C14:0	6.68 ± 0.06 ^a^	6.19 ± 0.05 ^b^	6.2 ± 0.11 ^b^
C16:0	44.87 ± 0.30 ^c^	51.36 ± 0.24 ^b^	56.11 ± 0.21 ^a^
Unsaturated fatty acids (UFA)			
C16:1	6.22 ± 0.28 ^a^	5.42 ± 0.07 ^b^	3.21 ± 0.04 ^c^
C18:1	9.25 ± 0.20 ^a^	5.03 ± 0.07 ^b^	2.19 ± 0.04 ^c^
C18:2	1.16 ± 0.11 ^a^	0.62 ± 0.03 ^b^	0.11 ± 0.03 ^c^
Cyclic fatty acid (CFA)	28.25 ± 0.30 ^a^	27.42 ± 0.19 ^b^	27.6 ± 0.17 ^b^
Minor fatty acids	1.48 ± 0.07 ^c^	2.17 ± 0.08 ^b^	2.99 ± 0.13 ^a^

Different letters indicate the significant difference (*p* < 0.05).

## Data Availability

The data presented in this study are available upon request.

## Sample Availability

Samples of the compounds are available from the authors.
